# ART Regimen and Other Sociodemographics Do Not Affect Cytokine Expression in HIV Patients in Ghana

**DOI:** 10.1155/2019/2730370

**Published:** 2019-09-09

**Authors:** Samuel Essien-Baidoo, Dorcas Obiri-Yeboah, Yeboah Kwaku Opoku, Elvis Ayamga, Kevin Hodi Zie, Daniel Attoh, Evans Obboh, Anna Hayfron Benjamin, Justice Afrifa

**Affiliations:** ^1^Department of Medical Laboratory Science, School of Allied Health Sciences, University of Cape Coast, Cape Coast, Ghana; ^2^Department of Microbiology and Immunology, School of Medical Sciences, University of Cape Coast, Cape Coast, Ghana; ^3^Biopharmaceutical Laboratory, College of Life Sciences, Northeast Agricultural University, Harbin, China; ^4^Department of Maternal & Child Health, School of Nursing and Midwifery, University of Cape Coast, Cape Coast, Ghana; ^5^Scientific Research Centre, Second Affiliated Hospital of Harbin Medical University, Harbin, China

## Abstract

**Background:**

HIV infection is marked by the production of cytokines by infected cells and cells of the immune system. Variations in the levels of cytokine in HIV-infected individuals significantly impact the role of the immune system with the possibility to affect the course of HIV disease by either exacerbating or suppressing HIV replication.

**Aim:**

The study sought to investigate the effect of sociodemographic indices, clinical laboratory parameters, and ART regimen on Th1, Th2, and Th17 cytokines in HIV patients.

**Materials and methods:**

A total of two hundred (200) HIV patients on either the first or second line of ART were recruited into the study. Sociodemographic indices were collected using researcher-administered questionnaires. Serum concentrations of two major immune-promoting cytokines, IL-12 and IFN-*γ*, and immune-suppressive cytokines, IL-10 and IL-17, were measured using enzyme-linked immunosorbent assay (ELISA). *T*-test and chi-square were used to compare mean scores, while correlation (Pearson's correlation) and linear regression analyses were also performed with the statistical significance set at *p* < 0.05.

**Results:**

The mean age of the participants was (45.54 ± 0.7846) years with a greater proportion (84.5%) between 31 and 60 years. The mean interferon-gamma (INF-*γ*), interleukin- (IL-) 10, interleukin-12, and interleukin-17 were estimated to be 349.9 ± 8.391 pg/ml, 19.32 ± 0.4593 pg/ml, 19.23 ± 0.3960 pg/ml, and 24.6 ± 0.6207 pg/ml, respectively. Although INF-*γ* and IL-17 levels were relatively higher in males compared to females, it was vice versa for IL-10 and IL-12. However, none of these was statistically significant. Again, no significant difference was observed among all the cytokines stratified by the duration of ART, stage of HIV, and smoking status. Most importantly, stratification by either first- or second-line ART regimens recorded no significant difference in cytokine levels. Age significantly correlated inversely with IFN-*γ* (*r* = −0.27, *p* ≤ 0.001), IL-10 (*r* = −0.24, *p* ≤ 0.001), and IL-12 (*r* = −0.18, *p*=0.01) while duration on ART significantly correlated inversely with IFN-*γ* (*r* = −0.16, *p*=0.02). CD4 counts at 6 months and 12 months on ART correlated inversely with IL-17 (*r* = −0.17, *p*=0.02) and plasma viral load at 1 year (*r* = −0.22, *p* ≤ 0.001), respectively. A positive correlation was observed between IFN-*γ* and IL-12 (*r* = −0.84, *p* ≤ 0.001) and IL-17 (*r* = −0.50, *p* ≤ 0.001). This positive trend was repeated between IL-10 and IL-12 (*r* = −0.92, *p* ≤ 0.001) and IL-17 (*r* = −0.61, *p* ≤ 0.001).

**Conclusion:**

The levels of IFN-*γ*, IL-12, IL-17, and IL-10 are not significantly affected by sociodemographics and ART regimen. This observation shows that no significant difference was observed in cytokine levels stratified by ART regiments. This means that both regimens are effective in the suppression of disease progression.

## 1. Introduction

Globally, the human immunodeficiency virus (HIV) infection and its accompanying sequelae, the acquired immune deficiency syndrome (AIDS), account for over 35 million deaths since the first reported cases in 1983 [1]. Infection with human immunodeficiency virus (HIV) impacts both directly and indirectly on systemic and local innate immunity. Inarguably, both innate and adaptive immune responses during primary HIV infection are crucial in the establishment of initial host immunologic control of viral replication [2]. Individuals with swift disease progression are characterized by greater T-cell activation and turnover, high viral load, and elevated levels of inflammatory cytokines [3]. Antiretroviral therapy (ART), the standard treatment of HIV infection, entails a combination of three or four drug sets including nucleoside reverse transcriptase inhibitors (NRTIs), integrase inhibitors (INIs), nonnucleoside reverse transcriptase inhibitors (NNRTIs), protease inhibitors (PIs), and fusion inhibitors (FIs) as the first line of treatment [4]. However, in case of treatment failure, a switch to a second-line ART regimen of a ritonavir-boosted protease inhibitor plus two NRTIs is recommended [5]. Global efforts towards universal access to ART have led to an increase in the number of patients on ART in both low- and middle-income countries [6]. ART coverage increased from about 3 million persons in 2007 to 9.7 million in 2012 [7, 8], resulting in substantial reductions in HIV-related morbidity and mortality globally [9]. Notwithstanding the promising clinical, immunological, and virological impact of HIV-infected patients receiving first-line ART [10, 11], countless patients are failing first-line ART and hence necessitating the switch to second-line ART [12, 13]. In sub-Saharan Africa, there is the necessity for about 6% of patients receiving first-line to switch to second-line ART regimens in any given year [8].

In HIV infection, infected cells and cells of the immune system produce myriads of cytokines [14]. These cytokines end up controlling the immune function and ultimately affect the replication of the virus [15]. Variations in cytokine levels in HIV-infected individuals immensely affect the function of the immune system with a consequent effect on the course of HIV disease progression by either enhancing or suppressing HIV replication [16]. Again, the course of HIV progression to AIDS can be significantly affected by disparities in cytokine production, leading to deregulation of the immune system [14]. The early phase of HIV infection is characterized by Th1 predominant cytokines such as IL-2 and interferon-gamma (IFN-*γ*) with the late phase characterized by a shift in Th1 to Th2 cytokines such as IL-4 and IL-10, TNF-*α*, and proinflammatory cytokines (IL-1, 6, and 8) [17]. Again during HIV infection, perturbation of Th17 cells which produces IL-17 at mucosal sites could add to the pathogenesis of HIV either by amplifying the susceptibility to bacterial and fungal infections or by unsettling the mucosal immune defenses [18]. However, ART partially reverses the abnormal cytokine profile contributing to the suppression of HIV replication and restoration of CD4 T-cell counts [19].

Cytokine profiles in ART are very significant in the monitoring of the clinical improvement and progression of the disease, especially in developing countries such as Ghana. We, therefore, sought to elucidate the impact of sociodemographic and clinical laboratory variables on cytokine profiles of HIV patients on the first- and second-line ART. This will be essential for clinicians to monitor the progression of the disease in patients on either regimen of ART.

## 2. Materials and Methods

### 2.1. Study Design/Study Site

A convenient cross-sectional study was employed to recruit HIV patients on ART into the study conducted at the HIV clinic within the Cape Coast Teaching Hospital (CCTH) located in the Central Region of Ghana. The HIV Clinic in CCTH serves as a referral hospital in the Cape Coast Metropolis serving patients within and beyond the Central Region in the capacity as a Teaching Hospital. Being the first and largest HIV clinic in the region, the clinic has a relatively large number of clienteles and averagely serves about 120 clients every week.

### 2.2. Eligibility/Exclusion Criteria

Individuals with autoimmune or some other form of immunosuppressive or immune-proliferative diseases were excluded from the study.

### 2.3. Study Population

All experimental protocols for the study were approved by the University of Cape Coast Institutional Review Board. Also, written consent was sought from the participants before enrolment into the study. A total of two hundred (200) HIV patients on ART aged 18 years and above attending the HIV clinic were enrolled in the study using systematic sampling after meeting the inclusion criteria. Sociodemographic indices were obtained using researcher-administered questionnaires. Clinical data of participants were collected from their clinical records under a data sharing agreement with the National AIDS/STI Control Programme (NACP). This information included the duration of HIV diagnosis, last WHO clinical stage before initiating ART, duration on ART and the ART regimen, CD4 counts at baseline, six months, and twelve months on ART, and plasma viral load after six months and after one year on ART.

### 2.4. Blood Sample Collection

Four milliliters of venous blood was collected into heparinized tubes and centrifuged for 30 minutes at 3000 ×g at 2°C to 8°C, within 30 minutes after collection. Aliquots of plasma were taken and stored at −20°C to −80°C. Samples were then assayed not more than two weeks after storage using the enzyme-linked immunosorbent assay (ELISA).

### 2.5. CD4+ T-Cell Count

Determination of CD4+ T-cell count was carried out at the Department of Microbiology Laboratory of the CCTH, Ghana, using 50 *μ*l of patients' samples. The test was run using the fluorescence-activated cell sorter (FACS) count flow cytometer (Becton Dickinson and Company, San Jose, USA) with strict adherence to the manufacturer's protocols and instructions.

### 2.6. Estimation of Viral Load

COBAS AmpliPrep/COBAS TaqMan HIV-1 v2.0 test (Roche, Switzerland) with an analytical sensitivity of 20 HIV-1 RNA copies/mL and 100% of specificity was used for the quantification of viral load using EDTA plasma (1000 *μ*l) with strict adherence to the manufacturer's protocols and instructions.

### 2.7. ELISA and Measurements of Cytokines

Reagents for the assay procedures were prepared and standard and sample wells set. 50 *μ*l of standards was added to standard wells and duplicated. 10 *μ*l of testing samples was added to sample wells, followed by 40 *μ*l of sample diluent. 100 *μ*l of HRP-conjugate reagent was added to each well, covered with an adhesive strip, and incubated for 60 minutes at 37°C. The wells were aspirated followed by washing five times each with 400 *μ*l of washing solution. After washing, the remaining wash solution was removed and the plates were inverted and blotted against clean paper towels. Chromogen solution A (50 *μ*l) and chromogen solution B (50 *μ*l) were added to each well, mixed, and incubated for 15 min at a temperature of 37°C shielded from light. Stop (50 *μ*l) solution was added to each well changing the color of the contents of the wells from blue to yellow. The optical density of the wells (OD) at 450 nm was read using a microtiter plate reader within 15 minutes. The concentrations of the individual samples were estimated using a standard curve of the OD of the known standard concentrations. The assay was repeated for interferon-gamma, interleukin-10, interleukin-12, and interleukin-17.

## 3. Data Analysis

Data were entered into Microsoft Excel Spreadsheet 2016, validated, and analyzed using GraphPad Prism version 7.01 for Windows (GraphPad Software, San Diego, CA, USA). Mean scores were compared by either *t*-test or chi-square test. Correlation (Pearson's correlation) and linear regression analyses were also performed. A *p* value <0.05 was deemed statistically significant.

## 4. Results

A total of 200 HIV participants on ART were recruited into the study with female preponderance (165 (82.5%)). The average age of the study subjects was estimated to be (45.54 ± 0.7846) years with the majority (84.5%) being between 31 and 60 years, urban dwellers (107 (53.5%)), and nonsmokers (198 (99.0%)). A greater proportion of the females were younger (44.81 ± 0.8452), cohabitating (66 (40.0%)), unskilled (131 (79.4%)), Christians (153 (92.7%)), and had no or little education (89 (53.9%)). Stratification of the sociodemographic variables by gender was significant for age, marital status, educational status, employment, and religion (Table 1).

Majority of the study participants had been on ART for more than four years and were still on first-line ART regimen (186 (93%)). The mean CD4 counts at baseline, six months on ART, and twelve months on ART were 220.8 ± 10.83 cells/mm^3^, 388.5 ± 13.29 cells/mm^3^, and 592.3 ± 15.27 cells/mm^3^, respectively. Participants who achieved plasma viral load less than 1000/ml after six months and 12 months on ART were (124 (62%)) and (182 (91%)), respectively. However, stratification of the clinical laboratory characteristic by gender did not reveal any significant differences (Table 2).

**Figure 1:**
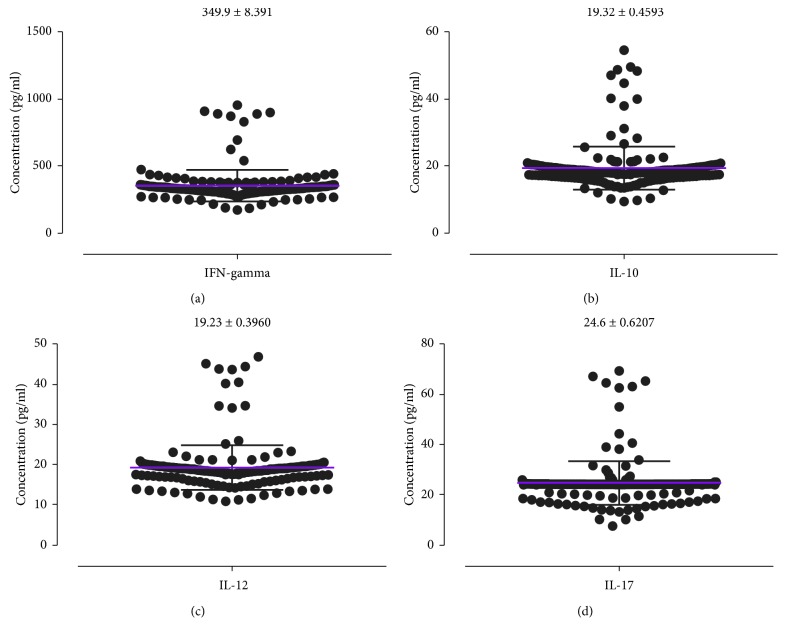
Distribution of cytokines among the study participants.

**Figure 2:**
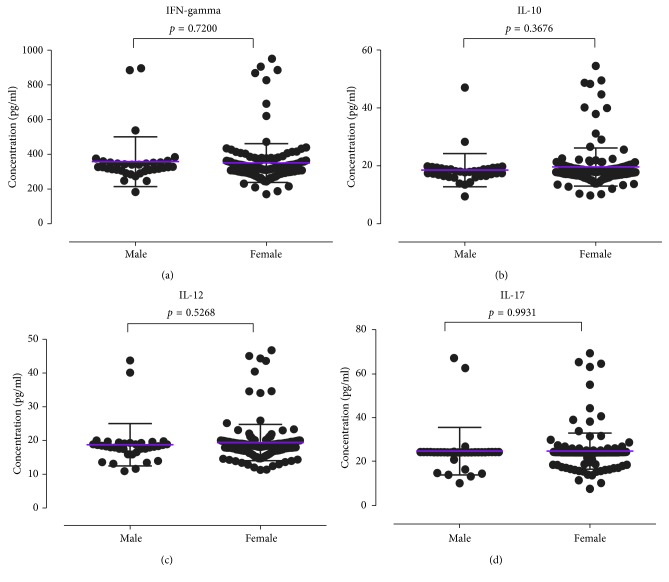
Cytokine distribution of the study participants stratified according to gender: (a) IFN-gamma, (b) IL-10, (c) IL-12, and (d) IL-17.

**Figure 3:**
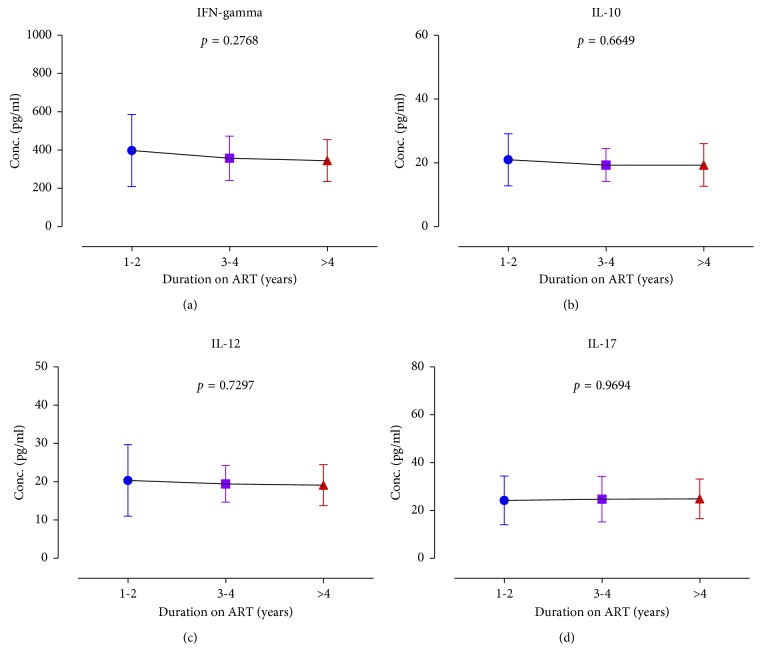
Cytokine distribution of the study participants according to their duration on ART: (a) IFN-gamma, (b) IL-10, (c) IL-12, and (d) IL-17.

**Figure 4:**
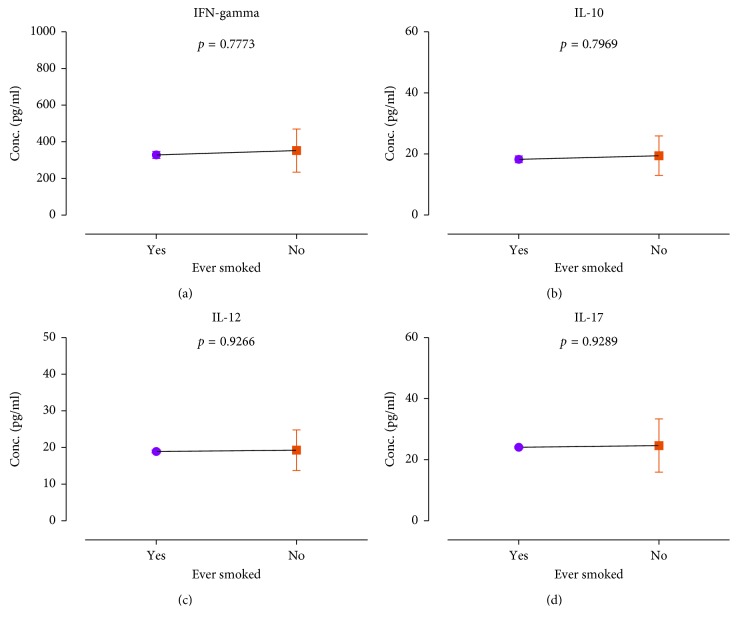
Association of smoking with cytokine distribution: (a) IFN-gamma, (b) IL-10, (c) IL-12, and (d) IL-17.

**Figure 5:**
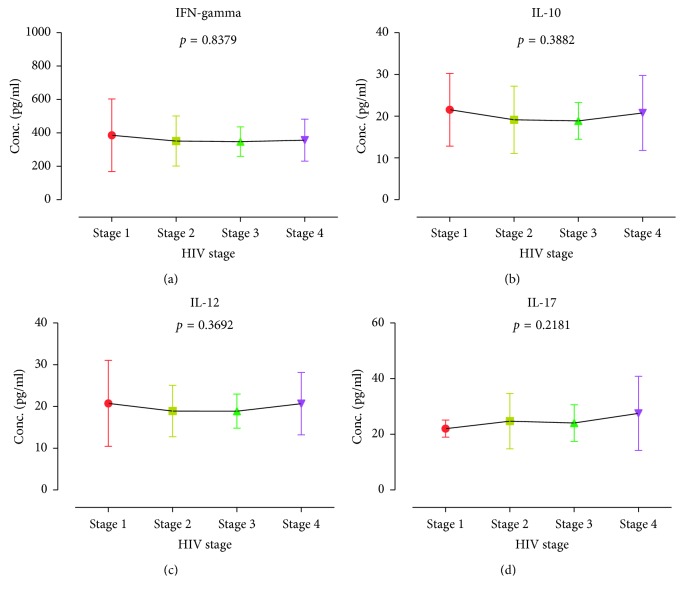
Cytokine distribution of the study participants according to the stage of HIV: (a) IFN-gamma, (b) IL-10, (c) IL-12, and (d) IL-17.

**Figure 6:**
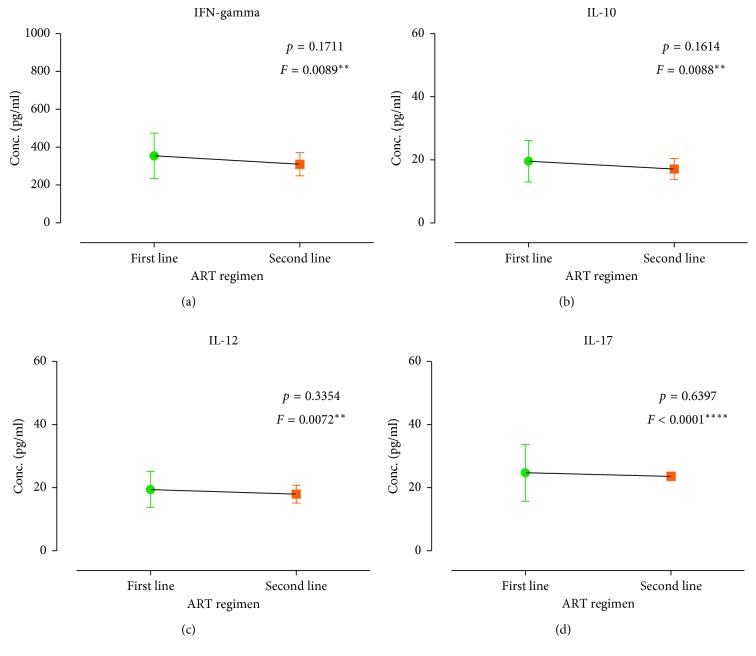
Cytokine distribution of the study participants according to the ART regimen: (a) IFN-gamma, (b) IL-10, (c) IL-12, and (d) IL-17.

The mean INF-*γ*, IL-10, IL-12, and IL-17 were estimated to be 349.9 ± 8.391 pg/ml, 19.32 ± 0.4593 pg/ml, 19.23 ± 0.3960 pg/ml, and 24.6 ± 0.6207 pg/ml, respectively (Figure 1). The cytokine distributions among study participants were further stratified by gender (Figure 2), duration on ART (Figure 3), smoking status (Figure 4), HIV stage (Figure 5), and ART regimen (Figure 6). INF-*γ* and IL-17 levels were relatively higher in males (356.5 ± 145.1 and 24.61 ± 10.93) than in females (348.5 ± 112.8 and 24.60 ± 8.291), while IL-10 and IL-12 were relatively higher among females (19.51 ± 6.642 and 19.34 ± 5.443) than among males (18.42 ± 5.761 and 18.68 ± 6.351) (Figure 2). INF-*γ*, IL-10, and IL-12 declined with the duration on ART, while IL-17 remained stable with the duration on ART (Figure 3). No smoking was associated with a slight elevation of INF-*γ*, IL-10, IL-17, and IL-12 (Figure 4). Stratification of the cytokines by stage of HIV infection revealed a declined from stage 1 through to stage 3 and an upsurge in stage 4 for INF-*γ*, IL-10, and IL-12 whereas IL-17 increased with stage of HIV (Figure 5). However, stratification of all the cytokines by the duration on ART, stage of HIV, smoking status, and most importantly by either first- or second-line ART regimens was all not statistically significant.

Sociodemographic and clinical laboratory indices correlated with cytokine levels. Age significantly correlated inversely with IFN-*γ* (*r* = −0.27, *p* ≤ 0.001), IL-10 (*r* = −0.24, *p* ≤ 0.001), and IL-12 (*r* = −0.18, *p*=0.01) and insignificantly with IL-17 (*r* = −0.00, *p*=0.98). Duration on ART significantly correlated with CD4 counts at 12 months (*r* = 0.16, *p*=0.03) and inversely with IFN-*γ* (*r* = −0.16, *p*=0.02). CD4 counts at 6 months and 12 months on ART correlated inversely with IL-17 (*r* = −0.17, *p*=0.02) and plasma viral load at 1 year (*r* = −0.22, *p* ≤ 0.001), respectively. A positive correlation was observed between both IFN-*γ* and IL-12 (*r* = −0.84, *p* ≤ 0.001) and IL-17 (*r* = −0.50, *p* ≤ 0.001). This positive trend was again repeated between IL-10 and IL-12 (*r* = −0.92, *p* ≤ 0.001) and IL-17 (*r* = −0.61, *p* ≤ 0.001) (Table 3).

## 5. Discussion

Despite the significant increase in access to ART in low- to middle-income countries, considerable reports of first-line ART failures have been recorded necessitating a switch to second-line ART. This observation can be partly attributed to the nonadherence to treatment guidelines by patients [20]. This study aimed at identifying differences and possible associations in the immunological profile, with the prognosis of the disease and sociodemographic indices in HIV+ patients under both the first- and second-line ART regimens.

In this study, the majority of the patients were within the age of 30–60 years with females occupying a more significant percentage. Sociodemographic data reveal a significantly higher number of participants with none to a secondary level of education, unemployed, and unskilled with females forming the larger proportion. A similar observation was observed in Tanzania with high HIV prevalence among those with low to no education [21]. Education plays a crucial role in mitigating the effects of HIV/AIDS by providing the knowledge base that informs self-protection and promoting behavior that lowers infection risks [22]. The number of unemployed and unskilled participants was significantly higher. Occupation directly translates into the socioeconomic status of an individual. In a study in Nigeria, an inverse association was observed between HIV prevalence and socioeconomic status [23]. This could, therefore, account for the observed high prevalence among this group. Stratification of all clinical laboratory characteristics of participants such as duration of diagnosis, duration on ART, ART regiment, and viral load by gender was however not statistically significant.

Immune activation, characterized by polyclonal B-cell activation, accelerated T-cell turnover, dendritic cell depletion, and elevated proinflammatory cytokine levels, is a hallmark of HIV infection [24]. The dysregulation of cytokines, essential markers of immune activation, has been shown to contribute to viral replication and disease progression [25]. Immune responses are raised during HIV infection causing CD4+ T helper cells to secrete cytokines, which regulate the immune response. Plasma cytokine levels give an intimation of the nature of immune response as well as an assessment of a patient's response to antiretroviral treatment [26]. Hence, cytokines such as IL-2 and IFN-*γ* secreted by CD4+ T cells during HIV infection are expected to be low whereas an upregulation in the production of T helper 2 (Th2) cytokines such as IL-4 and IL-10 and proinflammatory cytokines such as IL-1, 6, and 8 is expected [17]. IFN-*γ* activates macrophages and is indispensable for eliminating intracellular pathogens [25]. This shift has been labeled as a change from an environment characterized predominantly by Th1 cytokines which are associated with cell-mediated immune responses to Th2 cytokines known to promote humoral immune responses.

The low level of IL-10, which is a Th2 cytokine, and IL-17 shows retardation in the progression of the diseases attributable to ART as *in vitro* studies have demonstrated disease progression with excess production of Th2 cytokines such as IL-10 [27]. The role of IL-17 has been mainly linked to inflammatory conditions and autoimmunity [28] as stimulation by IL-17 leads to the second wave of different proinflammatory cytokines and chemokines such as GM-GSF causing the recruitment of neutrophil [29]. IL-17 again inhibits virus-induced cell apoptosis, thereby protecting virus-infected cells, and the inhibition of apoptosis further leads to increased life of infected cells which reduces cell death by cytotoxic T cells culminating into enhanced viral replication and persistence [30].

T helper (Th) cells have been categorized into two subgroups, i.e., Th1 and Th2, with a different pattern of cytokine profiles [31]. Th1 cytokines are characterized by secretion of IL-2, IL-12, and IFN-*γ*. These cytokines are associated with a protective response. On the other hand, the Th2-specific cytokines, i.e., IL-4, IL-5, IL-6, IL-10, and IL-13 [32], are associated with disease progression and lead to the progression of HIV infection to AIDS [33]. A swing from a Th1 to a Th2 cytokine pattern has been previously hypothesized to parallel or even precede the progressive CD4 impairment that accompanies the transition towards the final stage of AIDS [34]. In this study, the high IFN-*γ* and low IL-10 show a slow progression of the disease.

Although interferon-gamma and interleukin-17 levels were relatively higher in males than in females while interleukin-10 and interleukin-12 were relatively higher among females than among males, none of these reached a statistical significance. Again, no significant difference was observed among all the cytokines stratified by the duration on ART, stage of HIV, and smoking status. Most importantly, no significant difference was found in cytokine levels stratified by first- and second-line ART regiments. This observation shows that both regimens are equally effective in the suppression of the disease progression. Our findings are in tandem with the works of Keating et al. [35] and Amirayan-Chevillard et al. [36] but in contrast with that of Akase et al. [37].

Much is known about the maturation, aging, and senescence of the human immune system and the clinical ramifications of these processes [38]. Age significantly correlated inversely with IFN-*γ*, IL-10, and IL-12. Similar observations were made by Pettiford et al. with regard to IFN-*γ* [39]. Duration on ART significantly correlated with CD4 counts at 12 months and inversely with IFN-*γ*. CD4 counts at 6 months and 12 months on ART correlated inversely with IL-17 and plasma viral load at 1 year, respectively. This resonates with a cohort study on HIV patients' pool from Manhattan and Washington [25]. This research reports the importance of knowledge of cytokine expression by clinicians to monitor the progression of HIV infection. This can be done by a careful analysis of the interplay and shifts in the expression of Th1 and Th2 cytokines. The study has also clearly shown that the type of ART regimen administered does not significantly affect the expression of cytokines, an indication of equal effectiveness. Patients can therefore be assured of their health irrespective of their ART regimen. Therefore, any observed abnormality in cytokine expression in a patient on either ART regimen could be attributed to other factors such as an undiagnosed infection. Hence, an abnormal cytokine profile will therefore require swift attention and medical diagnosis.

## 6. Conclusion

In summary, the high level of IFN-*γ* coupled with the low level of IL-10 in our study participants shows retardation in the disease progression. Moreover, both the first and second lines of ART regimens used by our participants are equally effective in preventing disease progression.

## Figures and Tables

**Table 1:** Sociodemographic characteristics of HIV patients stratified according to gender.

Variable	Total	Male	Female	*p* value
*Age (years)*	45.54 ± 0.7846	48.94 ± 1.982	44.81 ± 0.8452	**0.0452** ^t^
≤30	14 (7.0)	1 (2.9)	13 (7.9)	0.2561^c^
31–60	169 (84.5)	29 (82.9)	140 (84.8)	
>60	17 (8.5)	5 (14.3)	12 (7.3)	

*Marital status*				
Single	54 (27)	6 (17.1)	48 (29.1)	**0.0017** ^c^
Married	73 (36.5)	22 (62.9)	51 (30.9)	
Cohabitating	73 (36.5)	7 (20.0)	66 (40.0)	

*Educational status*				
None to primary	96 (48.0)	7 (20.0)	89 (53.9)	**0.0003** ^c^
Up to secondary (senior high)	93 (46.5)	23 (65.7)	70 (42.4)	
Tertiary	11 (5.5)	5 (14.3)	6 (3.6)	

*Employment status*				
Unemployed	29 (14.5)	2 (5.7)	27 (16.4)	**0.0018** ^c^
Unskilled	157 (78.5)	26 (74.3)	131 (79.4)	
Skilled	14 (7.0)	7 (20.0)	7 (4.2)	

*Place or residence*				
Rural	93 (46.5)	12 (34.3)	81 (49.1)	0.1107^c^
Urban	107 (53.5)	23 (65.7)	84 (50.9)	

*Religion*				
Christian	182 (91.0)	29 (82.9)	153 (92.7)	**0.0056** ^c^
Muslim	16 (8.0)	4 (11.4)	12 (7.3)	
Traditional	2 (1.0)	2 (5.7)	0 (0.0)	

*Smoking*				
Yes	2 (1.0)	1 (2.9)	1 (0.6)	0.2241^c^
No	198 (99.0)	34 (97.1)	164 (99.4)	

Values are presented as frequency (percentages), mean ± SED; ^t^Student's sample *t*-test; ^c^chi-square test; *p* < 0.05.

**Table 2:** HIV-associated clinical and laboratory characteristics of the study participants stratified according to gender.

Variable	Total	Male	Female	*p* value
*Duration of HIV diagnosis (months)*	60.42 ± 2.998	57.71 ± 7.403	60.99 ± 3.286	0.6787^t^
<24	41 (20.5)	8 (22.9)	33 (20.0)	0.6735^c^
24–60	77 (38.5)	15 (42.9)	62 (37.6)	
>60	82 (41.0)	12 (34.3)	70 (42.4)	

*WHO staging*				
1	8 (4.0)	0 (0.0)	8 (4.8)	0.5214^c^
2	49 (24.5)	9 (25.7)	40 (24.2)	
3	114 (57.0)	22 (62.9)	92 (55.8)	
4	29 (14.5)	4 (11.4)	25 (15.2)	

*Duration on ART (years)*				
1–2	13 (6.5)	3 (8.6)	10 (6.1)	0.4895^c^
3–4	50 (25.0)	11 (31.4)	39 (23.6)	
>4	137 (68.5)	21 (60.0)	116 (70.3)	

*ART regimen*				
First-line regimen	186 (93)	32 (91.4)	154 (93.3)	0.6883^c^
Second-line regimen	14 (7)	3 (8.6)	11 (6.7)	

*CD4 count cells/mm* ^*3*^				
Baseline mean	220.8 ± 10.83	205.9 ± 22.51	223.9 ± 12.25	0.5283^t^
Mean after 6 months on ART	388.5 ± 13.29	339.2 ± 24.86	399.5 ± 15.16	0.0798^t^
Mean after 1 year on ART	592.3 ± 15.27	571.1 ± 32.80	596.4 ± 17.11	0.5434^t^

*Plasma viral load after 6 months on ART*				
0	51 (25.5)	8 (22.9)	43 (26.1)	0.4527^c^
1–999	73 (36.5)	16 (45.7)	57 (34.5)	
≥1000	76 (38.0)	11 (31.4)	65 (39.4)	

*Plasma viral load after 12 months on ART*				
0	40 (20.0)	9 (25.7)	31 (18.8)	0.5388^c^
1–999	142 (71.0)	24 (68.6)	118 (71.5)	
≥1000	18 (9.0)	2 (5.7)	16 (9.7)	

Values are presented as frequency (percentages), mean ± SED; ^t^Student's sample *t*-test; ^c^chi-square test; *p* < 0.05.

**Table 3:** Correlation of the sociodemographic, CD4 counts, plasma viral load, and cytokine distribution of the study participants.

		Age	HIV duration	ART duration	Baseline CD4	CD4, 6 months	CD4, 1 year	PVL, 6 months	PVL, 1 year	IFN-*γ*	IL-10	IL-12	IL-17
Age	*r*		0.17	0.14	−0.11	−0.02	−0.03	−0.22	−0.06	−0.27	−0.24	−0.18	−0.00
	*p*		**0.01**	**0.04**	0.12	0.77	0.68	**0.01**	0.36	**0.00**	**0.00**	**0.01**	0.98
HIV duration	*r*			0.47	−0.03	0.14	0.07	0.07	−0.03	−0.13	−0.11	−0.12	−0.07
	*p*			**0.00**	0.66	0.05	0.37	0.41	0.65	0.07	0.13	0.09	0.29
ART duration	*r*				0.03	0.13	0.16	0.05	0.07	−0.16	−0.09	−0.11	−0.03
	*p*				0.63	0.08	**0.03**	0.51	0.34	**0.02**	0.19	0.14	0.65
Baseline CD4	*r*					0.68	0.50	−0.07	−0.04	−0.01	−0.02	−0.07	−0.04
	*p*					**0.00**	**0.00**	0.42	0.58	0.86	0.76	0.32	0.56
CD4, 6 months	*r*						0.68	−0.03	−0.07	−0.03	−0.05	−0.09	−0.17
	*p*						**0.00**	0.71	0.36	0.73	0.52	0.23	**0.02**
CD4, 1 year	*r*							−0.07	−0.22	−0.09	−0.07	−0.08	−0.11
	*p*							0.38	**0.00**	0.24	0.32	0.29	0.16
PVL, 6 months	*r*								0.04	0.10	0.07	0.06	−0.02
	*p*								0.65	0.21	0.42	0.47	0.79
PVL, 1 year	*r*									0.06	0.04	0.05	0.02
	*p*									0.42	0.49	0.45	0.77
IFN-*γ*	*r*										0.84	0.84	0.50
	*p*										**0.00**	**0.00**	**0.00**
IL-10	*r*											0.92	0.61
	*p*											**0.00**	**0.00**
IL-12	*r*												0.63
	*p*												**0.00**
IL-17	*r*												
	*p*												

## Data Availability

All data used and analyzed in this study will be deposited at the archives of the Department of Medical Laboratory Sciences, University of Cape Coast, Ghana.
